# Editorial: New frontiers in parallel robotics

**DOI:** 10.3389/frobt.2023.1282798

**Published:** 2023-09-08

**Authors:** Sébastien Briot, Jessica Burgner-Kahrs

**Affiliations:** ^1^ Laboratoire des Sciences du Numérique de Nantes (LS2N), UMR 6004, Centre National de la Recherche Scientifique (CNRS), Nantes, France; ^2^ Department of Mathematical and Computational Sciences, University of Toronto, Toronto, ON, Canada

**Keywords:** parallel robot, continuum robot, cable-driven manipulator, underactuated robot hand, parallel manipulator

Rigid-link fixed-base parallel robotics is a mature research area and an enabler for developing various industrial applications (e.g., pick-and-place manipulation, machine tools, simulators, etc.). Fundamental scientific questions for these robots are now solved, leading to decreased scientific interest. On the other side, we have seen the research community’s rising interest in exploring new types of parallel robots, such as aerial parallel robots or parallel continuum or soft robots. Such new parallel robots can be exploited for novel application areas as they are lighter and more flexible while maintaining their traditional counterparts’ high precision and dynamics.

With this Research Topic, we are highlighting the frontiers of parallel robotics research, transcending the traditional definition and at exploring the new contours of parallel robotics. It focuses on emerging types of parallel manipulators that leverage soft or continuum robots, flying robots, cable-driven robots, underactuated robots, multi-finger hands, micro-scale parallel robots, etc. These novel closed-chain mechanisms, with no rigid links, fixed base, or traditional end-effector, raise significant scientific challenges.

We are also fully convinced that these emerging Research Topic can only be investigated adequately with bringing together researchers from those distinct communities but with a common interest in parallel robotics to share their experiences, methods, challenges, and issues. Addressing the challenges associated with these new Research Topic from several different complementary perspectives and solutions related to the design, modeling, and control of these novel complex solutions will be beneficial for accelerating the research on these new frontiers of parallel robotics.

This Research Topic, “*New Frontiers in Parallel Robotics*,” includes papers on cable-driven parallel robots (CDPRs), continuum parallel robots and underactuated grasping. Improving the performance of CDPRs is an essential in the community. Many current works are related to increasing their wrench closure workspace, studying the stability, proposing new control algorithms for the cable-tension allocation or improving their accuracy. In this Research Topic, we have two papers related to this Research Topic. The first one, entitled “*Cable failure tolerant control and planning in a planar reconfigurable cable-driven parallel robot*” Raman et al. is willing to propose an algorithm for control and planning which provides the ability to the robot to detect a cable break, thus improving the safety of its use by an operator in a collaborative context. The second paper, “Reconfiguration strategy for fully actuated translational cable-suspended parallel robots” Bettega et al. deals with the problem of conserving the tension into the cables while achieving a task in a large workspace of several meter length.

Continuum parallel robotics is an emerging field at the frontiers of continuum robots and parallel robotics. Many research questions are still open, among which are investigating new design concepts and finding efficient modelling, in particular for analyzing their dynamics, defining proper algorithms for their workspace computation or defining adequate indices for analyzing their performance. The paper “*Singularity analysis of 3-DOF planar parallel continuum robots with constant curvature links*” Lilge et al. belongs to the latter case. This work conducts a singularity analysis for all possible continuum parallel robot architecture with three identical legs, providing a geometrical understanding of singularity occurrences.

Underactuated robotics grippers are of great interest for many applications, especially for grasping fragile objects or avoiding the high cost of creating a robotized hand. The last (but not least) paper of this Research Topic, entitled “*A compact underactuated gripper with two fingers and a retractable suction cup*” Courchesne et al. presents the mechanical design of a new versatile and compact gripper able to grasp objects in constrained environments.

In conclusion, the research on parallel robotics has moved beyond the traditional rigid-link fixed-base robots, and the focus has shifted toward exploring emerging types of parallel manipulators. This shift has led to significant scientific challenges, including designing, modeling, and controlling novel closed-chain mechanisms. The Research Topic “*New Frontiers in Parallel Robotics*” highlights some of the recent research in cable-driven parallel robots, continuum parallel robots, and underactuated grasping. The papers in this issue propose new control algorithms, reconfiguration strategies, and singularity analysis, among others. These findings will accelerate research on the new frontiers of parallel robotics and enable the development of novel applications.

